# A review of computational models of basic rule learning: The neural-symbolic debate and beyond

**DOI:** 10.3758/s13423-019-01602-z

**Published:** 2019-05-28

**Authors:** Raquel G. Alhama, Willem Zuidema

**Affiliations:** 1 Language Development Department, Max Planck Institute for Psycholinguistics, Wundtlaan 1, 6525 XD Nijmegen, The Netherlands; 2 Institute for Logic, Language and Computation, University of Amsterdam, Science Park 107, 1098 XG Amsterdam, The Netherlands

**Keywords:** Rule learning, Statistical learning, Computational models, Neural-symbolic learning

## Abstract

We present a critical review of computational models of generalization of simple grammar-like rules, such as ABA and ABB. In particular, we focus on models attempting to account for the empirical results of Marcus et al. (*Science, 283*(5398), 77–80 1999). In that study, evidence is reported of generalization behavior by 7-month-old infants, using an Artificial Language Learning paradigm. The authors fail to replicate this behavior in neural network simulations, and claim that this failure reveals inherent limitations of a whole class of neural networks: those that do not incorporate symbolic operations. A great number of computational models were proposed in follow-up studies, fuelling a heated debate about what is required for a model to generalize. Twenty years later, this debate is still not settled. In this paper, we review a large number of the proposed models. We present a critical analysis of those models, in terms of how they contribute to answer the most relevant questions raised by the experiment. After identifying which aspects require further research, we propose a list of desiderata for advancing our understanding on generalization.

## Introduction

One key feature of human cognition is our capacity to discover regularities in the environment and to generalize them to novel cases. Crucially, generalization goes beyond exploiting perceptual similarities. A well-known example, introduced by Fodor and Pylyshyn (1988), is that an individual who understands the sentence *John loves Mary* can also understand *Mary loves John*^1^. To achieve that, an individual must abstract from the concrete properties in the input, and decide the extension of novel items to which the abstracted regularity or *rule* applies.

In experimental linguistics, and concretely in the tradition of Artificial Language Learning (ALL), generalization of structural combinatorial relations from language-like input is known as *rule learning*^2^. In the last two decades, a great body of experimental work has emerged, focusing on how humans discover relations that range from identity rules (Marcus et al., 1999; Gerken, 2006; Endress et al., 2007b) to nonadjacent dependencies (Peña et al.,, 2002; Gómez, 2002, 2005; Endress & Bonatti, 2007a; Frost & Monaghan, 2016) and even finite state grammars (Gomez & Gerken, 1999).

This experimental work is complemented with computational modeling studies, which use computational methods to formalize, implement, and simulate hypotheses about the cognitive mechanisms that operate during the experiments. One study that has become a particular focus for computational modeling work is that of Marcus et al., (1999): while reporting successful generalization of grammar-like rules by 7-month-old infants, it claimed that a large class of connectionist models, which were widely used for the study of language learning (and cognition more generally), could not solve this simple task.

Marcus et al.’s paper immediately caught the attention of the field, and a great number of computational modeling studies were presented in the years that followed the original publication. These models aimed to explain the original results, but in addition, they addressed the key question of what is required in order to generalize to novel items. Thus, a heated but contentive debate arose, and today, in spite of the recent revival of neural network models, this fundamental question is still not settled (e.g., see Calvo and Symons 2014; Lake & Baroni 2018; Marcus^2018^).

## Motivation, goals, and scope

In the debate about *whether* connectionist models can reproduce these results, important questions about *how* children solved the task received less attention. The goal of this paper is to revisit this debate, distill what the presented studies have shown about rule learning, and identify what still requires more investigation. It is not our goal to conclude the review with a choice for the “best” model(s); instead, we aim to learn lessons from the variety of presented models^3^.

Despite the vast existing literature on experimental work on rule learning, in this paper we focus exclusively on the Marcus et al. study, for several reasons. First, this study uses very simple rules, involving a repetition on a certain position in a syllable sequence, which can be thought of as basic to accomplish generalization in any domain. Second, even though these rules are very naturally formalized in symbolic models, neural network modelers did not find consensus on what is needed for the latter class of models to learn these rules.

Our work should facilitate navigating the abundant literature following up on the Marcus et al. study, and analyzing the main contributions therein. To that aim, we identify the main categories of models (Table 1). Note that some models could be classified in more than one category; we have chosen to classify the models according to the properties that allow for the clearest presentation of the similarities and contrasts between models, and of the types the arguments put forth in the debate.

**Table 1:** Overview of the reviewed models

Neural networks	Simple recurrent networks	Baseline: Simple recurrent network (Marcus et al., 1999)
		Analog encoding (Negishi, 1999)
		Segmentation (Christiansen & Curtin, 1999)
		Categorization (Seidenberg & Elman, 1999a)
		Transfer Learning (Altmann & Dienes, 1999)
		Prior experience (Altmann, 2002)
	Non-sequential	Autoencoder with Cascade Correlation (Shultz, 1999)
		Autoassociator (Sirois et al., 2000)
	With repetition detector	Positional Binding (Shastri & Chang, 1999)
		PLAYPEN (Gasser & Colunga, 2000)
		Abstract Recurrent Network (Dominey & Ramus, 2000)
Symbolic Models		Bayesian Lexical Model (Frank & Tenenbaum, 2011)

As can be seen in Table 1, the number of models of each class is very uneven, with neural networks dominating the discussion—although some of these neural models may be considered hybrid symbolic-connectionist. This is not a choice, but a reflection of the existing literature. This is probably due to the simplicity of the Marcus task, which is almost trivial for symbolic models (although symbolic models can help address relevant questions, as we argue later).

In the remainder of this paper, we describe the empirical findings from the experiment by Marcus and colleagues, and then jump to describing and critically analyzing the existing models. We then identify the contributions of the modeling work and organize them around four main questions. Our overview allows us to determine the progress made on different aspects of perception, representations and learning, but also to recognize that the evaluation criteria used in the different studies hamper model comparison and occasionally fail to show success in the task. Finally, we outline an agenda for further research, with concrete desiderata for future models.

## The original study

The experimental study presented by Marcus et al., (1999) investigated the acquisition of grammar-like rules by 7-month-old infants. Designed as an ALL experiment, this work uses language-like stimuli consisting of ‘words’ sampled from a manually designed artificial language. The strength of this method is that the properties of the artificial language can be carefully manipulated by the researchers, and thus languages are created conforming to the regularities that are being investigated—which can be statistical relations between syllables, prosodic patterns, dependencies, or any other type of structural information of interest. In this way, researchers can incorporate the structures under study in the stimuli while minimizing the presence of other cues, in order to avoid confounds.

The authors ran a total of three experiments, in which the participants were first familiarized with a short speech stream, and then tested on a different set of speech stimuli. The familiarization stimuli were created according to a certain ‘rule’ (which we will also call ‘pattern’ or ‘grammar’). For instance, in one condition, the familiarization stimuli consisted of triplets of syllables underlying the “ABA” rule. A possible speech stream would thus be *li na li – ta la ta – ni la ni – ga gi ga – ...*, where the dashes denote silence gaps of 1 second). Crucially, all the grammars used for generating the stimuli (ABA, ABB and AAB) involve syllable repetition. Formally, this is an identity rule: what matters is not which particular syllables are used, but the fact that two syllables in certain positions are identical.

Participants were subsequently tested with mixed stimuli, containing triplets consistent either with the familiarization grammar or with another control grammar (e.g., ABB). This was done with the Head-turn Preference Procedure (HTPP), which takes as dependent measure the amount of time that infants direct their gaze to the sound speaker playing each test stimulus (for details, see Nelson et al., 1995). Thus, if infants learn the difference between the two grammars, the expected outcome is a significant difference in the looking times between the two grammar conditions. In order to test whether infants could learn an abstract rule (instead of memorizing the particular lexical syllables), the test stimuli were deliberately chosen to contain syllables that never appeared in the familiarization stream (e.g., *de ko de* for ABA, or *de ko ko* for ABB).

The first experiment familiarized a group of infants with speech stimuli generated with an ABA grammar, while another group was familiarized with an ABB pattern. The test stimuli were common to both groups of infants, and involved triplets from both grammars, but containing novel syllables. The complete stimulus set used in the experiment is listed in Table 2.

**Table 2:** Stimuli used in experiment 1 in Marcus et al., (1999)

	Familiarization				Test
ABA	ga ti ga	li ti li	ni ti ni	ta la ta	wo fe wo
	ga na ga	li na li	ni na ni	ta ti ta	de ko de
	ga gi ga	li gi li	ni gi ni	ta na ta	
	ga la ga	li la li	ni la ni	ta gi ta	
ABB	ga ti ti	li ti ti	ni ti ti	ta la la	wo fe fe
	ga na na	li na na	ni na na	ta ti ti	de ko ko
	ga gi gi	li gi gi	ni gi gi	ta na na	
	ga la la	li la la	ni la la	ta gi gi	
3x triplet (random order)					

The authors found a statistically significant difference in the amount of time that infants directed their attention to each test stimulus between grammar conditions: the stimulus items that were inconsistent with the familiarization grammar received more attention. At the very minimum, these results suggest that infants picked up some regularity that allowed them discriminate between items of each grammar. 4

However, the authors themselves identified a possible confound: the consonants in the stimuli appear always in voiced–unvoiced–voiced combinations. Since infants seem to be sensitive to voicing distinctions from the first months of life (Eimas et al., 1971), it is possible that the participants learned the voicing pattern instead of the abstract ABA or ABB rule. This pattern would certainly be a type of generalization, although it would relate phonetic features rather than ‘lexical’ items (syllables)^5^ . In order to rule out this possibility, a second experiment was performed with stimuli that controlled for these patterns, as shown in Table 3. The responses of this second experiment again showed a significant novelty preference, so the authors conclude that infants learnt the abstract rules.

**Table 3:** Stimuli used in experiment 2 in Marcus et al., (1999)

	Familiarization				Test
ABA	le di le	wi di wi	ji di ji	de di de	ba po ba
	le je le	wi je wi	ji je ji	de je de	ko ga ko
	le li le	wi li wi	ji li ji	de li de	
	le we le	wi we wi	ji we ji	de we de	
ABB	le di di	wi di di	ji di di	de di di	ba po po
	le je je	wi je je	ji je je	de je je	ko ga ga
	le li li	wi li li	ji li li	de li li	
	le we we	wi we we	ji we we	de we we	
3x triplet (random order)					

In spite of this control, yet another interpretation was possible: infants could have distinguished the two types of stimulus items based on the presence or absence of an immediate repetition. To test for this hypothesis, the authors carried out a third experiment with two grammars that contain an immediate repetition: ABB and AAB. In this case, the participants again showed a significant preference for novel stimuli, ruling out the alternative explanation. The authors conclude that 7-month-old infants are capable of abstracting simple grammar-like rules.

### Which mechanism?

What is the cognitive mechanism responsible for learning these regularities? Seminal results on Artificial Language Learning, and many other studies that emerged afterwards, have been explained on the basis of *statistical learning*, a domain-general mechanism that discovers regularities by keeping track of distributional properties of the input, such as co-occurrences and transitional probabilities (Saffran et al., 1996a; Saffran et al., 1996b; Aslin et al., 1998). Thus, one possibility would be that this is the cognitive mechanism at play during the experiment.

The authors, however, reject this hypothesis. Their main argument is that a statistical learning mechanism would track distributional properties between *observed* lexical items, and thus it could not account for the stimuli in the test set, the statistics of which would amount to zero. The authors claim that a cognitive mechanism of a different nature must be at play; concretely, a rule-based mechanism that operates over *variables* rather than observed items.

The proposed mechanism stores basic units from the input stream (in this case, syllables) into registers (variables), depending on their position in the triplet. Thus, each triplet is decomposed in three variables X, Y, and Z, and each of these variables represent the syllable appearing in each position: for instance, *lejile* would be represented as X=le, Y=ji, Z=le. According to this proposal, infants learn rules that relate these variables rather than their content: for instance, for stimuli in the ABA grammar, the objective rule is [XYZ such that X equals Z] (Table 4 lists the rules describing each grammar). Since rules relate variables rather than items, they can naturally be transferred to the unseen items in the test stimuli.

**Table 4:** *Algebra-like* rules corresponding to each grammar

Grammar	Rule
ABA	YZ such that X equals Z
ABB	YZ such that Y equals Z
AAB	YZ such that X equals Y

It is important to note that this proposal is lacking relevant details for a model of human learning. To analyze this point, it is useful to recall Marr’s levels of analysis (Marr, 1982). Marr suggests a topology for computational models of cognition, such that: i) *computational level* theories are concerned only with characterizing the problem (what is the goal and why) and the strategy to reach a solution, ii) *algorithmic* or *processing level* models propose a mechanistic account of the process and the representations in the cognitive system, and iii) *implementational level* approaches explain how the process and representations are physically instantiated.

The rule-based account offers a characterization of the problem: it suggests that the space of solutions involves relational rules over positional variables that represent syllables. Thus, this proposal has elements of a computational-level explanation, though a good account of the goal and the strategy to converge to a certain rule are missing. In turn, the use of *variables* may be taken as a representational component of a processing level model, but an algorithmic explanation is incomplete without a mechanistic account of the process that transforms the input into the output.

The work we review in this paper attempts to fill in these explanatory gaps. Upon the publication of the original work, a fierce discussion emerged, largely dominated by the proposal of many variants of neural network models. The predominance of these types of models is somewhat expected: connectionist models have great acceptance as implementations of (a form of) statistical learning, but in their standard form, they do not incorporate the *symbolic* components required for representing variables. Thus, much of the discussion centered on the question of whether symbolic representations are crucial to account for these results, and if so, how should they be incorporated in a mechanistic model. It was not until years later that other (symbolic) models provided a look at the results from another perspective, attempting to fill in the missing pieces of a computational level explanation.

In the midst of these discussions, other fundamental questions about what really distinguishes these mechanisms and what can be learned from the experimental results were explored in much less detail. Our work delves into these issues, but we first present a review of the different models that were proposed to account for the experimental findings.

## The neural network models

Neural network models, the defining class of models in *connectionist* theories of cognition (Churchland, 1980), are seen as an implementation of *statistical learning*. These models consist of a set of units (or artificial neurons) that communicate through weighted connections, the weights of which are optimized through a learning procedure to perform a certain task.

There exist many flavors or architectures of neural networks, varying in things like the topology of connections or the training procedure. For this review, we find it most convenient to distinguish between three main types: (i) simple recurrent networks, (ii) neural networks with non-sequential input, and (iii) neural networks equipped with a repetition detector.

### The simple recurrent network models

**Fig. 1:**
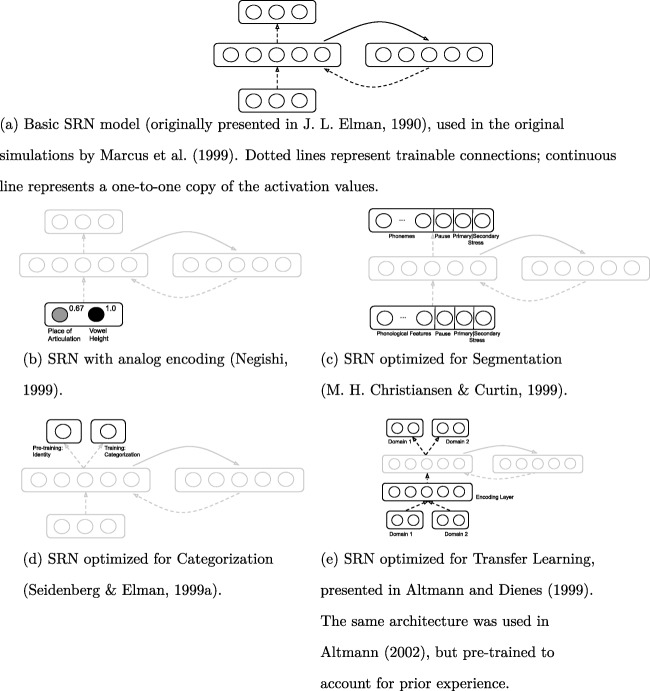
The SRN models. Figure 1a shows the basic SRN model used in the original simulations. In the variant SRN models, the part of the architecture that is common to the basic SRN is drawn in *lighter color*

The simple recurrent network (SRN) was proposed in Elman (1990) as a variant of the classic feed-forward network, specialized in learning regularities over *sequences*. The main contribution of this network is to process input data that unfolds through time. The architecture of an SRN (which can be seen in Fig. 1) incorporates a ‘context’ layer. At every time step, the activation values of the hidden nodes are copied into the context layer. The context layer is connected, through normal connections, to the hidden layer. The hidden layer thus reads activations from both the input layer and the context layer. Therefore, the internal representations depend on the previous state of the network, incorporating in this way a form of memory.

In their original study, Marcus et al. report simulations of their experiment with an SRN. In their work, the network was trained to predict the next syllable in the familiarization sequence. After some training, the model could perform well on the familiarization stream, but it did not yield good results with the test stimuli.

As mentioned before, the authors attributed this failure to the lack of symbolic operations, since this model does not explicitly encode variables. This argument had already been put forward for the broader case of generalization to novel input (Marcus, 1998), and it is consistent with the postulation of a specialized rule-based mechanism for generalization.

Marcus et al.’s interpretation proved controversial, however, and led many alternative computational models being proposed, as well as rivaling ideas about the nature of the rule learning mechanism. We now describe the proposed models, starting with those that maintain the architecture of the SRN but apply some variation.

#### An SRN with analog encoding

Negishi (1999) suggests that the lack of generalization in the original simulations is due to the fact that the encoding is based on *binary* activations. Instead, the author proposes to represent features of the input as real numbers; concretely, vowel height and place of articulation. The author replicated the original simulation with this encoding, obtaining larger prediction errors for the inconsistent test items—a fact that can be interpreted as reproducing the increased attention over inconsistent test stimuli observed in the experiment.

In a response to this study, Marcus (1999a) argues that the use of analog encoding can be seen as endowing the network with registers: if an input node represents all possible values, then it suffices to connect it to the output node with a weight of 1, and thus, the node would act like a variable that instantiates a particular value at a given time. (To us, this reasoning is not so convincing when applied to SRN models: the non-linearities in the hidden layer, and the connection with the recurrent layer do not permit the direct copy proposed by Marcus. As argued in Sirois et al., (2000), variable bindings are only effective if they can be accessed for further computation).

#### SRNs optimized for a different goal

### Segmentation

Christiansen and Curtin (1999) (and later also Christiansen et al., 2000) note that the same type of distributional knowledge that allows infants to perform speech segmentation at similar age (as attested by Saffran et al., 1996a) could be the basis for their success in the experiment reported by Marcus and colleagues. In other words, since infants are capable of tracking statistical properties that allow them to segment the input, these performed computations may also be helpful for the task proposed by Marcus et al. To test this hypothesis, the authors use an existing SRN model that learns to segment speech using different types of probabilistic information, presented in Christiansen et al., (1998).

This model is presented with a sequence of phonemes (instead of syllables), encoded with phonological features, primary and secondary stress, as well as whether the phoneme is the last one in a triplet (and therefore it is followed by a 1-s silence gap; see Table 5 for more details). The model is trained to predict an arbitrary representation of the next phoneme in the sequence, but also whether the phoneme is a syllable boundary, that is, whether it is followed by a 250-s silence gap (which is the length for pauses *within* triplets). In this way, the model is expected to learn to segment syllables after having been given the information about triplet boundaries.

**Table 5:** Encoding used in the neural network models reviewed

Models	Unit	Scheme	Features
Marcus et al. (1)	Syllable	Localist — Binary	
Marcus et al. (2)	Syllable	Distributed — Binary	6 phonetic features
Negishi	Syllable	Distributed — Analog	Place of articulation and
			Continuous vowel height
Christiansen&Curtin	Phoneme	Distributed — Binary	11 phonological features,
			primary and secondary stress,
			and presence of 1s gap
Seidenberg & Elman	Syllable	Distributed — Binary	12 phonetic features
Altmann and Dienes	Syllable	Localist — Binary	
Shultz	Triplet	Localist* — Analog	*Localist for syllables
Sirois et al.	Syllable	Localist — Analog	
Shastri&Chang	Syllable	Distributed — Binary	6 phonetic features
Gasser&Colunga	Triplet	Localist* — Binary	*Also includes an *angle*
Domeney&Ramus	Syllable	Localist — Binary	

The authors evaluate the model in two ways. First, they report that the network performs better at segmenting syllables belonging to triplets that are not consistent with the training grammar. The authors claim that the inconsistent items are therefore more salient, and this would explain why infants in the experiment pay more attention to inconsistent test items. Second, an analysis of the learnt internal representations is performed. The authors find that the representations for consistent and inconsistent triplets are distinguishable, as revealed by a two-group discriminant analysis.

To us, the task that this model is trained is somewhat unnatural: given that the speech stream is already segmented in syllable triplets with perceptible silence gaps, it is not clear why infants would additionally attempt to segment the stream into smaller units of the size of syllables. This fact is also observed by Marcus (1999a). In this letter, the author also argues that an analysis of the internal representations is not a suitable evaluation, since representations must have a causal effect on the output in order to be meaningful. Additionally, Marcus observes that the statistical significance of the analysis of the internal representations may not be meaningful, since the test consists of a very small number of items (four) compared to the number of hidden units (80) that provide the internal representation.

### Transfer Learning

The next model we review (Altmann and Dienes, 1999, based on an earlier model by Dienes et al., 1999), conceives of generalization as an instance of transfer learning between different domains. In the context of Marcus et al.’s experiment, the authors identify the domains as defined by familiarization stimuli and test stimuli. In order to account for the distinct domains, the authors extend the SRN architecture; concretely, the input and the output layers are augmented with extra nodes, such that two separate groups of nodes in each layer account for each domain. Additionally, the SRN is extended with an extra layer (the “encoding” layer), situated between the input and the hidden layer. The architecture of this network can be seen in Fig. 1e.

The network is first trained as a normal SRN, using only the input and output nodes of the first domain (D1). In the test phase, the stimulus is presented to the group of input nodes corresponding to the second domain (D2). Crucially, the test items are presented several times, and—contrary to the previously reviewed models—the network continues updating the weights, with the exception of those connecting the encoding layer with the hidden layer, which remain “frozen”. By keeping those weights intact, the model preserves some of the knowledge learned during training and attempts to transfer it to the test stimuli.

As explained in Altmann (2002), the encoding used in this model differs in one particular aspect: the representation of pauses. The authors use two different vectors to encode the pauses: one for the pauses that precede the onset of a triplet, and another to mark the ending. In other words, the silence gap between triplets is encoded as two consecutive pauses, the final pause for the previous triplet and the initial pause for the incoming triplet. In this way, the learning algorithm may detect different associations for onset and final syllables. We note that, besides unnaturally representing what perceptually is one single silence, this encoding provides the model with more explicit positional information than is available to competing models.

The authors measure the success of the network by computing the Euclidian distance between the predicted and target vectors, for the vectors resulting from the last iteration in the test. The results show higher correlations for prediction in the consistent grammar. Thus, it seems that this model managed to capture the regularity in the input: the frozen weights in the hidden layer presumably encode the learnt pattern, while the rest of the weights are no longer constrained by the training items, and thus allow for prediction of novel items. However, Marcus (1999c) observes that this implementation is consistent with the experimental results only when evaluating the results by computing the distance to the target. If, instead, one evaluates the most active unit in the predicted vector, then the model oscillates between the two grammars.

Additionally, it must be noted that in this model the domains are predefined, and there is no a priori reason for the test items to be part of a different domain. The model requires a mechanism that selects and freezes a subset of the weights, but it is not clear when and under what circumstances this mechanism would operate, and which particular subset of weights it should freeze. Thus, although this model was clearly useful for showing that freezing part of the weights and continuing learning only on another subset of the weights may be a necessary for generalization in neural networks, we agree with Marcus that it is not the final answer: the model is task-specific, and it remains unclear how it can be related to other cognitive mechanisms.

### Categorization

We now review a model that deviates from the original simulations in two ways: by changing the task into *categorization*, and by accounting for prior experience. Seidenberg and Elman (1999a) observe that the SRN presented by Marcus et al. had no previous knowledge, while infants in their experiment had been exposed to natural language in their environment. The authors argue that, by this prior exposure, infants might have learned to represent phonological similarity between syllables.

In order to account for prior knowledge, the authors extensively *pre-train* an SRN with 120 different syllables. In this pre-training phase, one single node is optimized to output whether the current syllable is the same as the previous syllable in the sequence. In this way, the SRN is trained to learn *identity* between syllables.

The weights learned during pre-training are used to initialize the SRN for the actual experiment, which is also defined as a categorization task, this time involving a different output node. Crucially, the network is not trained only with items belonging to one type of grammar (as the infants in the original experiment), but also with triplets generated from *both* ABA and ABB.

When tested with the novel triplets, the network shows responses close to zero for the ABA triplets and closer to 1 for ABB (concretely, 0.004 and 0.008 for *bapoba* and *kogako*, and 0.853 and 0.622 for bapopo and kogaga). Thus, it seems that the SRN learned to correctly discriminate between the grammars.

However, although the incorporation of pre-training is cognitively motivated, other aspects of this work require further justification, as discussed also in response letters (Marcus, 1999a, 1999b; Seidenberg & Elman, 1999b). The simulations greatly deviate from the original experiment in providing the model with negative evidence, and additionally, the incorporation of a feedback signal both during pre-training and training does not have its counterpart in the original experiment, since subjects in the experiment did not receive any form of feedback.

Marcus further observes that the output node is trained to follow the symbolic rule ‘if X==Y then 1 else 0’, suggesting that this evidences the need for symbolic operations. Although the model is clearly trained under that rule, as Seidenberg and Elman argue, the feedback is an external signal, which does not modify the space of hypothesis of the model. In other words, the fact that the supervision signal can be expressed with a symbolic rule does not entail that the network implements symbolic operations. It is nevertheless not clear where the signal for learning identity in the first place would come from, and whether it is plausible that a region of the brain is dedicated to finding identity relations in the input.

#### An SRN that accounts for previous experience

Before participating in the experiment, infants surely had been exposed to language in their environment. Altmann (2002) presents a study that accounts for prior knowledge by pre-training a model with natural language sentences. Concretely, the model is pre-trained to predict the next word in a sentence, for a set of 10,000 sentences generated from the grammar and vocabulary in Elman (1990). Note that words are encoded with a localist vector, without information about syllables or phonemes.

The architecture of the model and the encoding are the same used Altmann and Dienes (1999) (reviewed in “Transfer Learning”). In this study, the model is pre-trained as explained above, and then trained and tested with the Marcus et al. stimuli. Like in the previous proposals, the model is allowed to learn during the test phase, but in this study no connections are frozen, i.e., all the connection weights can be updated.

The output of the model is evaluated by computing the product moment correlation between the predicted vector and the target. An analysis of variance shows that consistent items exhibit a higher correlation. The authors conclude that their model reproduces the empirical data; however, the critiques that Marcus raised for previous work (Marcus, 1999c), based on the fact that the model iterates over the test items several times, apply also to these simulations.

### Non-sequential neural networks

We now review two neural network models that do not process the input stream sequentially, but instead, processes triplets separately (i.e., without taking into account the previous state of the network). These models are trained to find a suitable *representation* of the input: the objective function is set to minimize the error in reproducing the input pattern in the output layer, so the network needs to build representations in the intermediate layers that exploit the crucial features that allow for reconstruction in the output.

### An autoencoder trained with cascade-correlation

We first review the study presented in Shultz (1999) (later replicated in Shultz (2001) with a different encoding scheme). This model is implemented with an autoencoder architecture (Mareschal and French, 1997), which attempts to find a good representation (encoding) of the input. Unlike the SRN models, this network is trained with *cascade-correlation* (Fahlman & Lebiere, 1990), a training algorithm that gradually adds new nodes to the hidden layer, depending on the error (the difference between the model prediction and the input vector).

In the original simulations, each syllable is encoded with an arbitrary real number, which is represented in a single node. The information is presented to the network as triplets; thus, the input and output layers consist of three nodes, each one corresponding to one syllable. For the evaluation of the results, the author submits the error produced in the output layer to a repeated ANOVA. The results show a significant effect on grammar condition, with more error for inconsistent test items. Shultz concludes that this reproduces the original experiments, since more error requires further cognitive processing—as would be reflected in the increased looking times for inconsistent items.

Marcus (2001) argues that, due to using just one node to represent each syllable, the model can easily learn to copy the relevant syllables in the output. It is true that given the topology of the network, which is built *on the go*, it is easy to imagine that with the incorporation of a few nodes, the input gets roughly copied in the output (although distorted with the non-linear function applied to the hidden nodes), but (Vilcu and Hadley, 2005) showed with further analysis that the network does not perform such mapping.

Vilcu and Hadly further argue that these results are only replicable for this particular stimuli; for ABA or ABB sequences that involve different phonemes, the model is unable to distinguish between the grammars. Additionally, the authors show that the model does not generalize to stimuli encoded outside the range of the real numbers employed in the encoding.

It must be noted that this network operates over full triplets, creating in this way a somewhat artificial treatment of a continuous input. The next model we review incorporates a slightly more realistic treatment of time.

### An auto-associator model

Sirois et al., (2000) argue for a model that more clearly reproduces the experimental set up of Marcus et al., which involves habituation. The proposed model is as a fully connected neural network (i.e., all the nodes in the network are connected to each other); the external input is presented by activating a number of nodes, and the activity is propagated to all the nodes (including themselves) for a few cycles. The network is trained to reproduce this input, and in order to achieve this goal, it needs to strengthen the weights between nodes that have correlated activations, learning in this way associations in the input.

The input is processed as follows: each syllable in a triplet is presented to a different group of nodes, one at time; but crucially, the activation of the previous syllables is decreased (or ‘decayed’), creating in this way a fading memory of the input. Once the three syllables in a triplet have been presented, the activations of the network are reset. Thus, the model implements a certain representation of time, though only within triplets.

As for the evaluation, Sirois analyzes the number of presentations required for the test items to be assimilated (i.e., to be accurately reproduced by the network). The rationale is that a larger number of presentations would predict increased attention in infant behavior, since more cognitive resources would be required. The outcome is a significant difference between consistent and inconsistent items, so the model is taken to reproduce the original findings.

It must be noticed that the close reproduction of the original set up in this model is as much an advantage as a drawback: since the design is intimately tied to the experiment, the architecture would need to be adapted for even small changes of the stimuli. For instance, a simple variation of the experiment that involved sequences of syllables of non-uniform length (e.g., triplets and quadruplets) would not be easy to implement with this architecture, since the number of nodes required is determined by the number of syllables. This is a clear disadvantage with respect to the SRN models, which are flexible in this regard.

### Neural networks with a repetition detector

We now review three models that have a dedicated mechanism to detect repetitions of syllables in the input.

### A neural network with positional binding

(Shastri & Chang, 1999) present a model of the Marcus et al. experiment that implements a form of dynamic binding (Hummel, 2011). The model, originally presented in (Shastri et al., 1993), is implemented as a neural network with two groups of dedicated nodes for the input: one group that represents the phonetic features for an input syllable, and another group with three nodes corresponding to each of the three positions in a triplet. The idea behind dynamic binding is that the nodes of the two groups activate in synchrony, and therefore the *coincident* activity can be exploited by the network in order to learn the abstract pattern in the input.

**Fig. 2:**
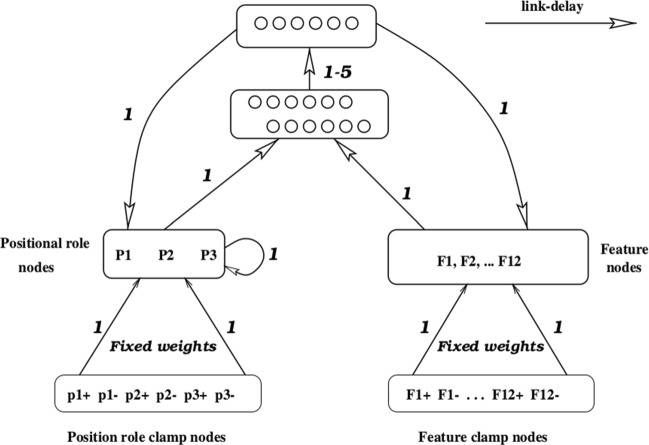
Model presented in Shastri and Chang (1999) (image courtesy of Lokendra Shastri). The *numbers* indicate link delays in the connections

This neural network model involves recurrent connections, but it is not implemented as a standard SRN. Instead, the model (illustrated in Fig. 2) clamps some input activations and propagates the activity through the network. After some delay, a target (the “correct” activation of the positional information) is clamped in the network. The difference between the actual activations and the target is used to update the weights through gradient descent.

Crucially, during the presentation of each syllable, all the positional nodes in which the syllable appears are active in the target; for instance, for the stimulus *ledile*, the first and the third positional nodes are *both* active on *each* presentation of *le*, and the second positional node is active during the presentation of *di*. It must be noted that, with the introduction of this form of feedback, the model is provided with an actual mechanism for detecting repetitions.

When it comes to the Marcus et al. experiment, the performance of the model is evaluated by computing the mean squared error between the model activations of the positional nodes and the target. The error is considerably smaller for test items consistent with the training grammar, and thus the model appears to reproduce the empirical findings.

Shastri and Chang argue that this approach offers a plausible mechanism to implement *rules* via biologically inspired temporal synchrony. Thus, this model is not presented as a counterargument to the claim by Marcus et al.; actually, Marcus (2001) reflects that this model implements *temporal* rather than *spatial* variables. However, as argued also in Shultz (2001), the design of the model is very tied to the actual experiment; additionally, the feedback is clearly unrealistic, in providing the model with the expected outcome rather than with the available information in the input. Therefore, we conclude that it does not yet offer a full reconciliation between symbolic and neural network models.

### The PLAYPEN model

Gasser and Colunga (2000) present a model (named PLAYPEN) that implements another form of dynamic binding. The authors formalize the task in the Marcus et al. experiment as extraction of correlations from the input: the authors emphasize that infants generalize to novel items because they learn *relational* correlations in the input, instead of content-specific associations.

**Fig. 3:**
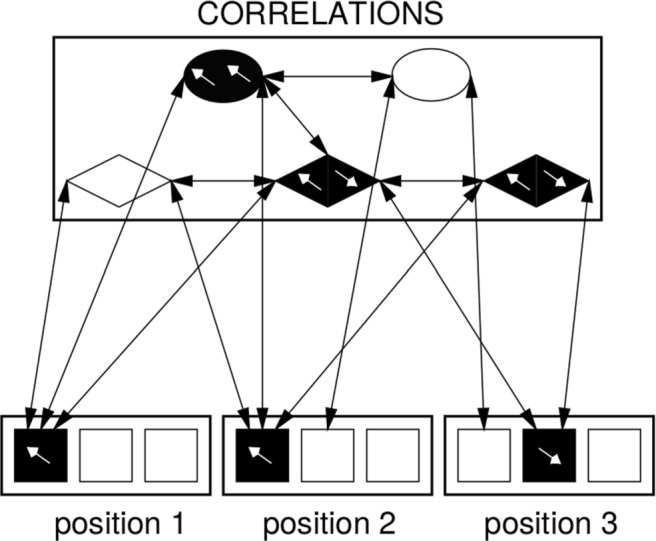
The PLAYPEN model (image courtesy of the authors, Gasser & Colunga 2000). *Diamonds* indicate difference relations; *ovals* indicate sameness

PLAYPEN is implemented as a generalized Hopfield network, that is, a fully connected neural network model in which weights are adjusted with the Contrastive Hebbian Learning algorithm (Hopfield, 1984). The network, illustrated in Fig. 3, is provided with dedicated units that detect sameness and difference. Therefore, its task is to reinforce the correlations according to the relations of sameness and difference found in the input.

As in the previous model, the authors assume dedicated nodes for each syllable position in a triplet. Additionally, the model is augmented such that each unit has an “angle”. The specific value of the angle is arbitrary, but it provides an additional dimension to the network, such that units with similar angle can be treated similarly. In this way, Gasser and Colunga implement a form of simplified dynamic binding.

The authors train ten instances of their model for each grammar condition. Afterwards, four repetitions of each test item are presented to the models by clamping the activation of the corresponding units. The average activation value of the relational units is stronger for the test items consistent with the training grammar, and the interaction between training and testing rule is significant(*p* < 0.001). Therefore, it appears that the network has strengthened the connections of the relations present in the familiarization stimuli.

In spite of the claims of biological plausibility of the model, its actual implementation remains extremely tied to the actual task, since the model comes with pre-wired relations over explicit bindings. Still, the authors argue that their model does not qualify as a symbolic model, since variables in symbolic models are content-independent, while PLAYPEN is sensitive to feature similarity between the presented items. However, while we agree that the model does not implement symbolic variables, it does incorporate rules, and thus it embodies the assumption that infants are equipped with a repetition detector.

### The abstract recurrent network

Dominey and Ramus (2000) simulate the Marcus et al. experiment with two models. Their first model is based on an architecture called temporal recurrent network (Dominey, 1995), a recurrent architecture in which only the weights connecting the hidden and the output layer are trained (while the rest are randomly initialized and remain unchanged). Interestingly, the nodes in this network are *Leaky Integrate-and-Fire* units: instead of single-valued activations, these units produce continuous *spikes*.

The authors do not find with this model a pattern of results that is consistent with the behavior of the infants in the experiment. Therefore, they simulate the experiment with an augmented version of the model, which they call abstract recurrent network (ARN). The ARN model features an additional short-term memory that stores the last five syllables of the input (in order to account for the 7 ± 2 *magical number* for short-term memory, Miller 1956), and a “recognition” component that detects whether any of the items in the short-term memory is the same as the actual item. This information is then provided to the internal state (the hidden layer), so that the model can exploit this information while updating the weights between the hidden layer and the output.

Given that the model is updated in continuous time, the authors measure the response latencies of the activation of the correct output nodes. These latencies should be smaller for learned items, since the strength of the activations in the network would influence the activity in the output. The authors find that statistically significant evidence: consistent stimuli items have shorter latencies, and this is taken to predict shorter looking times.

This model therefore appears to be a promising approach in incorporating successful learning with a more realistic treatment of time. However, as mentioned in the previous approaches, this network is endowed with a component that actively looks for repetitions in short-term memory (and even contains a node that fires when no repetition is found). This results in the very strong postulation that infants must be equipped with such a dedicated mechanism.

Importantly, this model adds some form of variables, in line with the claims by Marcus and colleagues. This is due to how the model accesses the augmented short-term memory, which is based on isolated memory positions dedicated to storing different items. It must be emphasized that this behavior comes from how the memory is *accessed*, not by the mere fact of adding short-term (spatial) memory; in other words, a network with this kind of memory may *learn* how to access it efficiently, and might eventually discover how it can exploit that nodes in the memory are dedicated to different items. This is not the case in the model by Dominey and Ramus, in which the recognition component is handcrafted to have positional access to elements in the short-term memory.

## The symbolic models

All the models reviewed so far are neural network approaches, though some of them incorporate some form of symbolic structure. To the best of our knowledge, the only existing symbolic models of the Marcus et al. experiment are Kuehne et al., (2000) and Frank and Tenenbaum (2011). As we reflect in “Which mechanism?”, a possible reason for this asymmetry with the number of neural network approaches is the simplicity of the grammars: when rules and variables are assumed, the task is so straightforward that there is not much room for unexpected findings. Nevertheless, in this section we review the latter model, since we think it offers a novel perspective onto the problem; concretely, it addresses the question of what determines which, among all the consistent rules, is preferred.

The Bayesian lexical model (henceforth, BLM; Frank & Tenenbaum, 2011) is designed to infer the symbolic rule that may have generated the observed data (i.e., the stimuli). The hypotheses space consists of combinations of rules that apply over syllables within triplets. Since the model is applied to a variety of datasets, this inventory is adapted to each experiment; in the case of the experiment by Marcus and colleagues, the hypothesis space incorporates an identity relation over syllables in the input. Therefore, the model may converge to any combination of identity relations that is consistent with the stimuli.

The model uses Bayesian inference to decide which is the most likely rule *r* (grammar) that may have generated the observed stream of triplets *T* = *t*_1_,...,*t*_*n*_; in other words, the goal of the model is to find for which rule *r* the *posterior* probability *p*(*r*|*T*) is maximal. As defined in Eq. 1, the posterior can be computed as a product of the *likelihood* of observing the data assuming this rule *p*(*T*|*r*) (see Eq. 2) and the *prior* probability of the rule *p*(*r*) (that is, the probability of a rule before observing the input stream). In order for the posterior to be a probability, this product needs to be normalized by the sum of the probabilities for all possible rules:

1 $$ p(r|T) = \frac{p(T|r)p(r)}{{\sum}_{r' \in R}p(T|r')p(r')} $$

where

2 $$ p(T|r) = \prod\limits_{t_{i} \in T}p(t_{i}|r), $$

and where

3 $$ p(t_{i}|r) = \frac{1}{|r|}. $$,

with |*r*| denoting the total number of triplets that are congruent with rule *r*.

The prior is defined as a uniform distribution; hence, each rule is *a priori* equally likely. As for the likelihood, the authors assume *strong sampling*, that is, the triplets are assumed to have been uniformly sampled from the set of triplets that a rule can generate. This entails that the probability of observing a triplet under a certain grammar is larger for “smaller” grammars (that is, grammars that generate a smaller number of triplets), as seen in Eq. 3. This creates a bias in the model in favor of more concise grammars; for instance, for a triplet such as *je-li-je*, a rule like ABA is more likely than a more general but equally consistent rule ABC that involves no identity. This bias is referred to as the *size principle*, and is consistent with infants behavior in similar experiments (Gerken, 2006, 2010).

Given the simplicity of the experiment and the model, the only rule that can compete with ABA or ABB is ABC, that is, a grammar which generates triplets consisting of three arbitrary syllables, which may or may not be repeated. This model shows that more probability is attributed to the more specific grammars ABA and ABB (consistent with the *size principle* defined above).

The authors present two additional variants of this model: one that assumes a certain amount of noise in the generative process (regulated through an additional parameter), and another that additionally allows for the possibility that the data was generated from multiple rules.

Due to the addition of parameters, the model now requires a procedure of *fitting*. The posterior probabilities derived from the model are related to the human responses through the use of the negative log probability (surprisal). These two variants also attributed more probability (less surprisal) to the rules involving an identity relation, although some small probability mass was attributed to the general rule ABC.

The BLM model thus identifies which rules would be favored under a rational model incorporating a bias for less general rules. Although the model is simple in comparison to the neural network approaches (mostly due to the fact that that the hypothesis space is relatively small and manually defined, ad hoc for the experiment), it brings an additional value that should not be underestimated. Concretely, it is the only model that clearly defines which biases are hypothesized to guide the preference for certain rules over others. Thanks to that, this approach incorporates a principle that postulates why the participants induced an identity rule instead of a more general rule in which all triplets are possible.

The model, however, is fiercely criticized by Endress (2013), questioning the validity of the *size principle* as a cognitive bias. The author reports an independent experiment in which participants are exposed to instances of an ABB grammar, using spoken syllables. The participants could discover at least two rules: the identity rule between the second and third syllable, or a more general rule glossed as “any sequence of human speech syllables”. In the subsequent test phase, participants had to choose between an ABB sequence of monkey vocalizations, or AAB triplets carried by human speech. The results show a preference for the AAB human triplets, a fact that is interpreted by Endress as contrary to the *size principle*. Frank (2013), in turn, argues that this data shows a modality preference rather than a rule preference. The issue is therefore not settled: it is not clear whether the subjects prefer certain generalizations based on the “size” of the set of items that a rule can generate, or based on a bias favoring acoustic aspects of the input (i.e., the vocalizations).

## Model evaluation

Although the models reviewed address the same experimental data, they greatly differ in the criteria applied to judge their success in reproducing the empirical phenomenon. The original experiment evaluates statistical significance over looking times, and this is taken as evidence for individuals having learnt the abstract rules. As is often the case in cognitive modeling, the proposed models are not evaluated in terms of reproducing the behavioral responses^6^, but rather, on their ability to learn some regularity in the input that allows for drawing a distinction between the two conditions.

In the original SRN simulations by Marcus and colleagues, it is not clear how the output of the model was evaluated, since the authors only report that “the network is unable to distinguish the inconsistent and consistent sentences”. In many of the follow-up papers, modelers use some error measure (e.g., mean squared error) to compute how much the predicted vectors deviate from the target (Negishi, 1999; Altmann and Dienes, 1999, 2002; Shultz, 1999; Shastri & Chang, 1999); generally this is accompanied with a statistical analysis that shows whether the computed error allows for distinction between grammars.

The rest of the reviewed studies apply other criteria. Sirois et al., (2000) compare the number of stimuli presentations required to succeed at generalization in each condition. Seidenberg and Elman (1999a) report the average activity of the output node in their model (trained for grammar classification). Gasser and Colunga (2000) also evaluate the average activity of certain nodes in the model—in this case, those that are dedicated to encode the rule–, and report the interaction between train and test rules. In contrast, Christiansen and Curtin (1999) and Christiansen et al., (2000) frame the problem as a segmentation task, and evaluate their models on the prediction of internal pauses within a triplet. Additionally, they analyze the internal representations built by the model, and find that they are distinguishable for each type of grammar. Dominey and Ramus (2000) takes the latency of output node related to the correct prediction as a proxy for looking times. Finally, Frank and Tenenbaum (2011) report whether the grammar preferred by their model is the one hypothesized by Marcus et al. (ABA or ABB, vs. other consistent grammars such as ABC).

In this paper, we argue that a new and consistent evaluation methodology is needed (see desideratum 6). There are two main arguments for this. First, given the variability of measures used to evaluate the models, it is not possible to compare them quantitatively. Second, we observe that there is a fundamental problem with most of these evaluation criteria: models are tested on some form of *discrimination* between items generated from each grammar, but not on whether they have learn the hypothesized rule (exceptions to this are the models in Shastri & Chang 1999, Gasser & Colunga^2000^, and Frank & Tenenbaum 2011). This stems from an ambiguity in the original results: the statistical significance between grammar conditions can emerge even if infants have not perfectly learned the hypothesized rule.

## Analysis of the models

In the previous section, we have reviewed models that appear to offer distinct perspectives on generalization. We now identify what we think are the most relevant questions that the Marcus et al. experiment raised, and analyze whether these seemingly contrasting approaches differ when answering those questions.

### Question 1: Which features or perceptual units participate in the process?

In the experiment, infants are exposed to a synthesized speech stream. The way this stream is perceived must impact what is learned from it, and therefore, details of this perceptual process are relevant. For instance, do infants perceive the input as a sequence of phonemes, or is the syllable the most salient perceptual unit? Do they analyze lower-level properties, such as phonetic features, once a syllable has been recognized? Do other acoustic dimensions, such as loudness or pitch, play a role in what is learned? How does the insertion of silence gaps affect the perception of the basic units?

These questions have not received much attention. However, thanks to using computer modeling, researchers are forced to make choices about how to represent the input and how to present it to the model over time. This is reflected in the encoding, which may, for instance, incorporate some detailed acoustic aspects of the stimuli or, instead, represent them with arbitrary symbols that do not encode any of the acoustic properties of the item. The latter is the approach taken by symbolic models such as the BLM, where each syllable is represented with an arbitrary symbol. However, in the case neural network models, the input vector can be coded in either way, as explained next.

In a localist encoding scheme, vectors are initialized to a null value (typically, 0 or -1) except for one of its units, which will have a non-null activation value (typically, 1). The position of the active unit indicates which item is represented, but it does not convey any information of the properties of the item; therefore, localist representations are always arbitrary. On the contrary, in a distributed encoding scheme, each unit may participate in the representation of more than one item. Although these values can be chosen arbitrarily, each unit may be chosen to represent one particular property of the item, and thus, the distributed vector would encode certain specific properties of the stimuli. As shown in Table 5, models vary in the choice of represented acoustic features.

The choice of the encoding scheme has an impact on generalization. As argued in Marcus (1998), for a standard neural network to succeed in generalizing to novel items, such novel items must fall *within the training space*. Marcus defines *training space* as the subset of the input space covered by the feature values (i.e., the value of each unit) of the training items. As an example, consider an encoding scheme based on three features [*f*_1_,*f*_2_,*f*_3_], and a training set consisting of the vectors [1, 1, 1], [1, 0, 0] and [1, 0, 1]. In this setup, a novel item like [1, 1, 0] lies within the training space, since all the features *f*_1_, *f*_2_ and *f*_3_ have appeared in the training data with values 1, 1, and 0, respectively. On the contrary, a novel item like [0, 1, 0] is outside the training space, since *f*_1_ = 0 did not appear during training, and consequently, a standard neural network model^7^ would never predict an item with *f*_1_ = 0.

For this reason, the choice of the encoding scheme and the dimensions to encode is relevant, since a certain amount of overlap is needed for generalization in standard neural networks. To illustrate this, consider the case in which localist representations are used. The nodes that represent the test items will be zero during familiarization, and therefore the learning algorithm will update the connecting weights until they converge to zero, so they would never be active to predict novel items. On the contrary, with distributed vectors, some of the units representing the test stimuli may have been active during training.

This raises two relevant issues. First of all, only distributed vectors stand a chance of yielding the desired generalization behavior, and whether they do depends on the overlap between vectors in training and test. This overlap can be accomplished in two ways: either by using pseudo-random symbolic initializations that guarantee a certain amount of overlap, or by investigating which are the relevant properties of the input that need to be coded in the vector. To our knowledge, this issue has also not been thoroughly explored. Thus, this will be one of our points in the desiderata for future work (1).

Second, the fact that a neural network may show only some degree of generalization (by predicting a vector that is *close* to the ‘correct’ vector) begs the question of whether infants in the experiment are exhibiting similar behavior. The behavioral responses show discrimination between grammars, but the experimental paradigm cannot distill whether infants would accurately predict the next syllable or would just have a close enough prediction to distinguish between test items. It is therefore not clear whether we should expect models to produce perfect generalization or a statistically significant difference in responses between grammars. For this reason, we propose that models are evaluated at least on both aspects, as we reflect later in the desiderata (see desideratum 6).

Finally, an aspect of the representation of the input that has not received enough attention is the treatment of time. Almost all the neural network models reviewed receive the input as discretized units, and update the weights of the model after each presentation (sometimes during a few timesteps, as in Shastri and Chang, 1999). The only exception is the spiking neural network model by Dominey and Ramus (2000), but even in this model we can find discrete syllable registers in its short-term memory. The use of discrete input has also forced the model to have a very unnatural representation of pauses, which are generally coded as one symbol—as if it were one more item in the vocabulary. For this reason, in the desiderata we suggest to investigate generalization over continuous input (desideratum 9).

### Question 2: What is the learning process?

One of the most ambitious goals behind the Marcus et al. experiment and modeling work is to shed light on the nature of the learning mechanism that is operating during the experiment. In neural networks, the implemented process of learning is commonly referred to as “associative learning”, and it is characterized by responding to contingency relations in the data. Most neural networks are trained with some form of gradient descent; in the majority of cases, the algorithm used is backpropagation (Rumelhart et al., 1988). Although the neurobiological plausibility of backpropagation has often been in questioned (Zipser & Andersen, 1988; Crick, 1989; Stork, 1989), later work argued that the learning procedure can be implemented also with biologically plausible bidirectional activation propagation (O’Reilly, 1996, 1998); moreover, Xie and Seung (2003) prove that—under certain conditions—backpropagation is mathematically equivalent to contrastive Hebbian learning, a process that is more commonly accepted as biologically plausible. All the neural network models reviewed implement some form of gradient descent, with the exception of the proposal of Dominey and Ramus (2000), which features a learning algorithm based on cortico-striatal circuits in the brain (Dominey, 1995), which researchers have interpreted as a form of least mean squares (Lukoševišius, 2012).

The rule-based model proposed by Frank and Tenenbaum (2011) learns through Bayesian inference over a predefined space of hypotheses. Therefore, this model does not offer an account of the process that induces the regularities in the first place. The authors clearly state that the model is proposed as an “ideal learner”: framed at Marr’s computational level, the aim of this proposal is not to account for the constraints of the human cognitive system, but rather to characterize the space of solutions and what would be the optimal solution given a certain prior. Therefore, a cognitively realistic rule-based model that explains *how* learning takes place during the experiment is still lacking.

### Question 3: Which generalization?

The speech streams we are concerned with are generated according to an ABA, AAB, or ABB pattern, which involves relations between *syllables*. However, the stimuli are also compatible with other rules, and—as discussed in *Question 1*—regularities may also appear in other acoustic dimensions.

In order to illustrate this, Table 6 shows some of the rules that describe the relations between syllables in the triplets. These can be as general as ‘*three consecutive syllables*’ (equivalent to rule (a)), or they could operate over two of the syllables in the triplet. These basic rules can be composed with logical operators (*and, or, not*), such as ‘*(a) and (b)*’; for instance, if a learner is hypothesized to learn a rule like ‘*triplet containing an adjacent repetition*’, this can be expressed as ‘*X=Y or Y=Z*’. As will be explained in the next section, theories that postulate that rules are cognitively real need to disambiguate which rule is being learned when rules are equivalent in their extension.

**Table 6:** Summary of some of the rules that the learners in Marcus et al. may extract

	ABA	ABB	AAB	Consistent
(a)	*X* *Y* *Z*	*X* *Y* *Z*	*X* *Y* *Z*	
(b)	*XYZ* : *CVCVCV* (*CV* : *Consonant* − *Vowel*)	*XYZ* : *CVCVCV*	*XYZ* : *CVCVCV*	
(c)	*XYZ* : *X*≠*Y*	*XYZ* : *X*≠*Y*	*XYZ* : *X* = *Y*	
(d)	*XYZ* : *Y* ≠*Z*	*XYZ* : *Y* = *Z*	*XYZ* : *Y* ≠*Z*	1,2,3
(e)	*XYZ* : *X* = *Z*	*XYZ* : *X*≠*Z*	*XYZ* : *X*≠*Z*	1,2
(f)	presence of repetition	presence of repetition	presence of repetition	
(g)	presence of nonadjacent repetition	presence of adjacent repetition	presence of adjacent repetition	1,2
(h)	voiced-unvoiced-voiced	voiced-unvoiced-unvoiced	voiced-voiced-unvoiced	1

These rules could further be composed with *and*, *or*, and *not* operators (e.g., [(*c*)]*AND*[(*d*)] suffices to explain the three experiments). Column “consistent” indicates whether the rules suffice to explain results in experiments 1, 2, and/or 3

From the models we have reviewed, only the Bayesian approach Frank and Tenenbaum (2011) explicitly tackles this question. In order to distinguish between otherwise equiprobable consistent hypotheses, this model incorporates a predefined rational principle that determines which hypothesis should be favored; in this case, the *size principle*. Therefore, the BLM provides a transparent way to test how different principles would be favored by a probabilistic inference process, an aspect that is missing in the neural network models.

It must be noted that rational principles are not the only source of disambiguation to decide between competing rules. For instance, Endress et al., (2005) report experimental evidence showing that repetition-based grammars are easier to learn when the repetition takes place in the edge positions. This entails that other aspects–such as perceptual factors—can also impose saliency in certain dimensions of the stimuli, breaking the uniformity between otherwise equivalent rules. For this reason, we suggest in desiderata 2, 3, and 4 that alternative factors that influence why certain rules are favored should be explored.

### Question 4: What are the mental representations created?

Another question raised by the experiment is how the induced rule is represented in the cognitive system. As researchers, we are used to employ formal languages for scientific descriptions, and thus it is natural to characterize stimuli with formal rules such as *XYZ such that X=Z*. And indeed, the behavior shown by the infants in Marcus et al. experiment can be described as following an identity rule that accounts for the familiarization grammar. However, the mental representations of infants in the experiment need not directly map with the components of such formal expression: the variables and the logical operators that relate them may or may not correspond directly to mental entities.

When it comes to representations, symbolic and non-symbolic models have different strengths and weaknesses. Symbolic rules can easily accommodate some of the most interesting properties of thought and language, such as systematicity, productivity and compositionality. The use of variables that are blind to the specific properties of their content allows for a binary output: rules are either consistent or inconsistent, never in between. However, this has the downside of endowing the models with little flexibility, and thus they are often not robust to noise (Opitz and Hofmann, 2015) 8. In contrast, neural network models do not explicitly represent rules or variables, so relations are content-dependent (as well as context dependent). One of the advantages of these models is that they can naturally account for degraded instances or accidental gaps; therefore, exceptions can be handled without the need of additional mechanisms (Elman, 1999).

As argued in Pylyshyn (1991), it is common in science that a debate arises when the object of research involves a system that can be easily described with rules. The author outlines a topology for theories addressing those type of systems, according to which the ontological status of the theory can be seen as a point in a spectrum between two extremes. Theories may, on one extreme, postulate rules that only account for regularities in the behavior of the system. In this case, rules function only as a theoretical descriptive construct, but the theory is agnostic towards the representations and the principles *followed* by the system. In intermediate positions, theories postulate that *some* of its elements correspond to principles or properties materialized in the system, and on the other extreme, theories maintain that all its rules and representations are explicitly encoded in the system. In the latter case, the elements in the canonical expression of a rule (including its symbols and the relations between them) correspond to certain properties in the system. Thus, details such as the total number of rules and their precise definition (e.g., whether they are based on identity or difference, even if their scope is identical) become relevant for a theory that posits that these rules are materialized in the cognitive system.

This characterization of theories can be easily related to Marr’s levels of analysis. The rule-based model in Frank and Tenenbaum (2011) is explicitly stated at Marr’s computational level; therefore, the fact that it employs symbolic rules is not to be taken as a representational claim. On the other hand, neural network models are implementational-level accounts of how processes and representations may be realized: unless the architectures incorporate additional symbolic mechanisms, the underlying claim is that the behaviour observed in the experiment can emerge even when symbolic representations are not employed.

## An agenda for formal rule-learning research

The previous analysis has allowed us to closely examine how this collection of models has helped in advancing our knowledge on the main research questions. Surprisingly, however, in spite of the relative simplicity of the experiment and the vast number of models, the state of our knowledge appears rather incomplete when we analyze the questions at this level of detail. For this reason, we have compiled an agenda of the issues that require more attention.

### **Desideratum 1**

Investigate which features should be encoded in the input representation, and quantify the overlap of features needed for generalization to occur.

To begin with, not much attention has been devoted to how the perception of the input can affect generalization. The syllables in the original experiment are chosen to minimize phonetic overlap, but as pointed out by McClelland and Plaut (1999), other acoustic cues may exhibit regularities. Additionally, similar experiments involving a different set of syllables show null results (Geambasu&Levelt, p.c.), suggesting that low-level cues might be relevant.

As mentioned before, the amount of overlap in the representation of input vectors in neural network models influences the prediction of novel items. Thus, more research is needed to quantify the amount of overlap required to reproduce the empirical findings, and specially, which features should be encoded in the vectors (and therefore, which perceptual dimensions guide generalization).

### **Desideratum 2**

Investigate perceptual biases.

The second issue that can be observed is that, in all models, the perceptual units (generally syllables) are treated equally, regardless of the position they appear at. However, as mentioned before, experimental work shows that syllables that appear in the edge of sequences are more salient to humans, to the extent that some rules are not learned if the regularity appears in middle positions (Endress et al., 2005).

The reviewed models do not explicitly incorporate any such biases. Although a case could be made for neural networks being able to *learn* those biases, this would only occur when saliency facilitates the task. Thus, we suggest that future efforts should be directed to investigate which perceptual biases facilitate or hinder generalization.

### **Desideratum 3**

Investigate the role of prior experience.

Most of the models reviewed are used as a *tabula rasa*: they are initialized with some independent method (e.g., randomly sampled weights in a neural network) and then trained exclusively on the familiarization data. However, for a randomly initialized model, it is unlikely that a short exposure to the familiarization stimuli suffices to reproduce the experiments. If, instead, the initial state of the models incorporates relevant prior knowledge, the learning procedure may converge more easily to the generalizing solution that infants seem to learn.

This is the idea behind the models proposed by Altmann (2002) and Seidenberg and Elman (1999a), but our analysis concluded that these models are not convincing explanations of the empirical phenomenon. Moreover, the use of pretraining procedures would be more explanatory if the they allow us to pinpoint which aspects of the prior knowledge are the ones influencing generalization to novel items.

### **Desideratum 4**

Model the coexistence and competition of rules.

It is very unlikely that information can be described with one single rule, but rather, an input stream is likely to incorporate coexisting regularities, possibly between different dimensions. A model of generalization should explain how the induced rules coexist, that is, how are multiple hypotheses represented and whether and how they relate or interfere with each other.

There are multiple factors that could influence the preference for some rules over others. As outlined in the desiderata above, perceptual factors and prior experience are some of the aspects that individuals may consider when they favor some rules over others. External factors, such as contextual information, may also play a role. As an example (adapted from Tenenbaum & Griffiths 2001), consider a math student who, after seeing numbers 30 and 40, is asked whether 41 or 50 are part of the same set. We would expect a preference for 50, as a result of inducing a mathematical rule such as ‘multiple of 10’. However, if we show these same numbers to a medicine student focused on the study of dangerous cholesterol levels, we expect her to generalize to 41 rather than 50, given that a rule based on proximity of these quantities is more relevant in this context. Thus, given the same input, context can change the favored generalizations.

From the models we have reviewed, only the BLM addresses this question, by assigning probabilities that weight coexisting hypotheses, and proposing a criterion (the *size principle*) to assign a preference for some rules over others (see also Chater and Vitányi (2003) for a more general argument for *simplicity* as a rational principle for disambiguating between equiprobable inductions). However, while (probabilistic) symbolic models are naturally suited for modeling this aspect of learning, the neural network models that we have reviewed do not offer any insight in this regard. Thus, one desideratum for neural network approaches to this problem is to explain which factors predict the preference for some rules over others, and propose an account of how rules coexist and compete.

### **Desideratum 5**

Incorporate independently motivated pressures for learning generalizing solutions.

In neural network models, the hypothesis space often contains multiple local optima, and thus the learning procedure has high risk of getting stuck in one of those. This entails that, in practice, there exist multiple solutions that may be found by a neural network model. While these solutions may be sufficient to account for the training data, they may not be *generalizing* solutions that can be transferred to the test stimuli.

This can be seen as a form of *overfitting*. Since neural networks have many degrees of freedom, they can easily find one of the non-generalizing solutions. In order to push a neural network model to find a generalizing solution, an additional source of pressure is needed. Thus, we believe an important avenue of future research is to investigate how to incorporate independently motivated biases to find generalizing solutions.

### **Desideratum 6**

Forge a consensus on evaluation criteria for models.

One of the most pressing issues that we have discovered in this review is that there is much disagreement in the formulation of the objective to optimize and, specially, on how to evaluate the outcome of a model. As is common in HTPP experiments, the original study compares the looking times between the two conditions, and this is taken as an indication that participants have learnt something that allows them to discriminate between conditions. However, the details of this response mechanism are not well known (though see Bergmann et al., 2013 for a model of the HTTP). Thus, having alternative evaluation criteria that help revealing what has been learnt can be helpful—but as we have observed, having too many of them hinders model comparison.

This is specially relevant for this experiment, since reflecting on the evaluation has revealed an important issue about the empirical data: while the results exhibit statistically significant difference in the attention that infants show between different grammars, there is no evidence that infants perform accurate generalization (i.e., correct prediction of the last syllable in a triplet), and this uncertainty is rather fundamental, as we do not know of experimental procedures that allow us to investigate what exactly have infants learned. Progress in understanding the latter would definitely facilitate that modelers settle for one particular evaluation method and compare the different model proposals in a systematic manner.

For lack of better knowledge, we suggest that models are evaluated on both criteria: i) whether they exhibit statistical significance for grammar discrimination, and ii) whether the model accurately generalizes to new items.

### **Desideratum 7**

Bridge the gap between levels of analysis: investigate how neural networks perform apparently symbolic computations.

As discussed before, one of the most debated issues is the ontological status of symbolic rules and variables. Even though the neural network models reviewed have arguably shown some success in reproducing the empirical findings, the relation between the symbolic-like behavior and its actual realization in the model is not completely clear: neural networks are assumed to perform symbolic-like computations implicitly, but how exactly this is done remains elusive. Thus, in order to understand how non-symbolic systems perform apparently symbolic computations, we should aim to investigate the internal representations and strategies employed by neural networks. Recent work on neural-symbolic integration has started addressing these issues (e.g., Alhama & Zuidema 2016, 2018, Alishahi et al., 2017; Hupkes et al.,^2018^).

### **Desideratum 8**

Models should learn spontaneously from brief or limited exposure.

Neural network models start from a largely unconstrained parameter space, and thus need to iterate over the data multiple times in order to converge to a good parameter setting. This is aggravated in the case of small training datasets (as would be the case for the Marcus et al. stimuli), since less data entails that more epochs are required for convergence. Unfortunately, this does not reproduce the training setup used in the infants experiment, in which the familiarization stimuli are presented only once. Although it could be argued that the procedure of iterating multiple times over the stimuli in the model reflects the availability of the data in short-term memory (such as the phonological loop) in the children, this does not allow us to distinguish between tasks that can be learned spontaneously and those that require a longer exposure or even a developmental trajectory.

For this reason, we think that achieving spontaneous learning with neural network models is an ambitious but relevant project. Recent advances on neural network architectures involving augmented memory (e.g., the neural Turing machines, Graves et al., 2014) are capable of fast memorization and retrieval of input stimuli, and thus they offer a promising avenue for learning from short exposures (e.g., successes in one-shot learning have been reported in Santoro et al., 2016). Research progress on constraining the initial state of the models based on prior knowledge (as outlined in desideratum 3) may also be beneficial for faster convergence to generalizing solutions.

### **Desideratum 9**

Investigate the effect of the continuous nature of speech in generalization.

An aspect that has been widely neglected in the reviewed models is to represent the continuous nature of speech. Some of the models operate over all the data at once (concretely, the Bayesian model), while the neural networks process the data in an incremental fashion, either over triplets, syllables or phonemes. But even in this case, the stream is pre-segmented, and the models are updated synchronously in discrete timesteps (as argued before, the model by Dominey and Ramus (2000) offers a more realistic treatment of time, but it does not succeed in modeling the experiment without pre-segmenting the input syllables and accessing its storage in a symbolic fashion).

By simplifying the representation of time, some aspects of auditory processing can be neglected. For instance, the speed at which an auditory speech stream is played may have an effect on the structural dependencies that learners can extract from it, due to the temporal proximity of the items involved (e.g., Wang et al., (2017) show that increasing the speech rate improves learning of non-adjacent dependencies). This phenomenon cannot be modeled with discrete neural networks, since there is no manipulation that can account for the speed of presentation of the syllables. It remains an open question whether a more realistic treatment of time would also bring new insights to the question of generalization.

## Conclusions

The study by Marcus and colleagues has been very influential in the field, thanks to showing generalization abilities in 7-month-old infants that had not been previously attested. It is fair to point out that these results have not always been so easy to replicate (see footnote 1 in Gerken (2006) as an example; also Geambasu&Levelt, p.c.); likewise, other generalization experiments (Gomez & Gerken, 1999) show behavior in the opposite direction, with infants looking significantly longer to consistent rather than inconsistent test items (as explained in footnote 4). Thus, unraveling under which conditions Marcus et al.’s study can be replicated requires further investigation, as well as a methodology to compare the outcomes of a group of related studies (e.g., see a methodological proposal for meta-analysis of experimental data in Tsuji et al.,^2014^). Nevertheless, even if we conclude that the experiment can only be replicated under very specific conditions, its design has undeniably been very fruitful for posing concrete questions about the nature of generalization.

In this work, we have contrasted different modeling traditions. Even though some approaches may appear irreconcilable at first glance, it actually seems that neural networks and Bayesian approaches are somewhat complementary in their contribution to understanding generalization. The former offer a theory of how humans may discover relevant regularities in the input at all, while the latter provide clear criteria for why they might prefer one generalization over another. In neural networks, it is difficult to predict beforehand which of the competing generalizations would be chosen, and thus, as explained in the previous section, the choices for the represented dimensions of the input will have an impact on what is learned. In contrast, in Bayesian models, we can pre-specify the hypothesis space and test rational principles to distinguish between possible generalizations, but the models lack the architectural constraints of learning that neural networks provide.

Overall, our study shows that, in spite of the many questions that can be raised from the Marcus et al. study and the large number of modeling contributions, most of the discussion has been centered around the question of the ontological status of rules and symbols. Although we agree that one of the most intriguing issues in cognitive science is to discover whether rule-like behavior requires symbolic operations, we hope that our review has highlighted other aspects of the problem that have received too little attention, such as the impact of perceptual factors and the question of which rule is preferred among competing consistent rules. Future experimental and modeling work on generalization in these directions would help in unraveling the foundations of this key aspect of human cognition.

## Footnotes

1 Fodor and Pylyshyn refer to this property of human thought as *systematicity*.

2 The term *rule learning* can be easily misinterpreted, since it appears with different meanings in the literature. In particular, it has also been used to postulate a specific theory of generalization (which we present later), according to which the knowledge extracted in such experiments must be explicitly represented as an algebraic symbolic rule. Unless otherwise specified, our use of *rule learning* is interchangeable with *generalization*; and *rule* is by default used to convey the same meaning as *pattern* or *regularity* unless it appears in the context of the specific theory that postulates algebraic rules.

3 The idea of revisiting this unsettled debate was partly inspired by the unpublished MSc thesis of Woensdregt (2014).

4 One possible explanation for the novelty preference is the *Goldilocks effect*: infants dedicate more cognitive processing to items that are neither too predictable, nor too unpredictable (Kidd et al., 2012). The inconsistent triplets in the test set can be thought of as items that meet this criteria, since they differ from the familiarization grammar but all follow the same, alternative pattern (the alternate grammar).

5 Both generalizations involve a repetition, but differ on the aspect that is repeated. In a voiced–unvoiced–voiced triplet, the voiced syllables may be different, as long as they incorporate a voiced phoneme. In contrast, ABA syllable sequences need not only preserve the voiced–unvoiced–voiced pattern, but also employ identical syllables in the A positions. Both patterns are an instance of a more general XYX rule, where X and Y represent either phonetic features or syllables.

6 Sometimes models are accompanied by *response models* that transform the model output into a measure that can be interpreted as a behavioral response, but this is not the case for the models we have reviewed.

7 The reason for this is that backpropagation updates the weights independently; in particular, the weights connected to an output node are updated independently to the weights connected to a different output node. Marcus refers to this aspect of the backpropagation algorithm as *training independence*.

8 Probabilistic symbolic models, like the BLM model, are sometimes deemed as alleviating the rigidness of symbolic models. However, note that, in the BLM model, probabilities are applied over the hypothesized rules, while the representation of the rules is still fully symbolic: a rule is either consistent or inconsistent with the stimuli.

## References

[CR1] Alhama, R. G., & Zuidema, W. (2016). Pre-wiring and pre-training: What does a neural network need to learn truly general identity rules?. In T. R. Besold, A. Bordes, A. d’Avila Garcez, & G. Wayne (Eds.) *Proceedings of the Workshop on Cognitive Computation: Integrating Neural and Symbolic Approaches (CoCo at NIPS2016)*. http://ceur-ws.org/Vol-1773/ (Vol. 1773 pp. 26–35).

[CR2] Alhama RG, Zuidema W (2018). Pre-wiring and pre-training: What does a neural network need to learn truly general identity rules?. Journal of Artificial Intelligence Research.

[CR3] Alishahi, A., Barking, M., & Chrupala, G. (2017). Encoding of phonology in a recurrent neural model of grounded speech. In R. Levy, & L. Specia (Eds.) *Proceedings of the 21st Conference on Computational Natural Language Learning (CoNLL 2017)* (pp. 68–378). Vancouver: Association for Computational Linguistics.

[CR4] Altmann GT, Dienes Z (1999). Rule learning by seven-month-old infants and neural networks. Science.

[CR5] Altmann GT (2002). Learning and development in neural networks—the importance of prior experience. Cognition.

[CR6] Aslin RN, Saffran JR, Newport EL (1998). Computation of conditional probability statistics by 8-month-old infants. Psychological Science.

[CR7] Bergmann, C., Ten Bosch, L., Fikkert, P., & Boves, L. (2013). A computational model to investigate assumptions in the Headturn Preference Procedure. *Frontiers in Psychology*, 4. 10.3389/fpsyg.2013.00676 PMC3790075 24109461

[CR8] Calvo P, Symons J (2014). The Architecture of Cognition: Rethinking Fodor and Pylyshyn’s Systematicity Challenge.

[CR9] Chater N, Vitányi P (2003). Simplicity: a unifying principle in cognitive science?. Trends in Cognitive Sciences.

[CR10] Christiansen MH, Allen J, Seidenberg MS (1998). Learning to segment speech using multiple cues: A connectionist model. Language and Cognitive Processes.

[CR11] Christiansen, M. H., & Curtin, S. (1999). Transfer of learning: rule acquisition or statistical learning?, (Vol. 3. ISSN 1364-6613. 10.1016/S1364-6613(99)01356-X. http://www.sciencedirect.com/science/article/pii/S136466139901356X 10.1016/s1364-6613(99)01356-x 10431257

[CR12] Christiansen, M., Conway, C., & Curtin, S. (2000). A connectionist single mechanism account of rule-like behavior in infancy. In *Proceedings of the T,wenty-second Annual Conference of the Cognitive Science Society*, (pp. 83–88).

[CR13] Churchland P (1980). A perspective on mind-brain research. Journal of Philosophy.

[CR14] Crick F (1989). The recent excitement about neural networks. Nature.

[CR15] Dienes Z, Altmann G, Gao S-J (1999). Mapping across domains without feedback: A neural network model of transfer of implicit knowledge. Cognitive Science.

[CR16] Dominey PF (1995). Complex sensory-motor sequence learning based on recurrent state representation and reinforcement learning. Biological Cybernetics.

[CR17] Dominey PF, Ramus F (2000). Neural network processing of natural language I. sensitivity to serial, temporal and abstract structure of language in the infant. Language and Cognitive Processes.

[CR18] Eimas PD, Siqueland ER, Jusczyk P, Vigorito J (1971). Speech perception in infants. Science.

[CR19] Elman JL (1990). Finding structure in time. Cognitive Science.

[CR20] Elman, J. (1999). Generalization, rules, and neural networks: A simulation of Marcus et. al. Html document.

[CR21] Endress AD, Scholl BJ, Mehler J (2005). The role of salience in the extraction of algebraic rules. Journal of Experimental Psychology: General.

[CR22] Endress, A., & Bonatti, L. (2007a). Rapid learning of syllable classes from a perceptually continuous speech stream. *Cognition*, *105*(2), 247–299. 10.1016/j.cognition.2006.09.010 17083927

[CR23] Endress, A., Dehaene-Lambertz, G., & Mehler, J. (2007b). Perceptual constraints and the learnability of simple grammars. *Cognition*, *105*(3), 577–614. 10.1016/j.cognition.2006.12.014 17280657

[CR24] Endress AD (2013). Bayesian learning and the psychology of rule induction. Cognition.

[CR25] Fahlman SE, Lebiere C (1990). The cascade-correlation learning architecture. Advances in Neural Information Processing Systems.

[CR26] Fodor JA, Pylyshyn ZW (1988). Connectionism and cognitive architecture: a critical analysis. Cognition.

[CR27] Frank M, Tenenbaum J (2011). Three ideal observer models for rule learning in simple languages. Cognition.

[CR28] Frank MC (2013). Throwing out the Bayesian baby with the optimal bathwater: Response to Endress. Cognition.

[CR29] Frost R, Monaghan P (2016). Simultaneous segmentation and generalisation of non-adjacent dependencies from continuous speech. Cognition.

[CR30] Gasser, M., & Colunga, E. (2000). Babies, variables, and relational correlations. In *Proceedings of the Twenty-second Annual Conference of the Cognitive Science Society*, (Vol. 22 p. 160): Lawrence Erlbaum Associates.

[CR31] Gerken L (2006). Decisions, decisions: infant language learning when multiple generalizations are possible. Cognition.

[CR32] Gerken L (2010). Infants use rational decision criteria for choosing among models of their input. Cognition.

[CR33] Gómez RL (2002). Variability and detection of invariant structure. Psychological Science.

[CR34] Gomez RL, Gerken L (1999). Artificial grammar learning by 1-year-olds leads to specific and abstract knowledge. Cognition.

[CR35] Gómez R, Maye J (2005). The developmental trajectory of nonadjacent dependency learning. Infancy.

[CR36] Graves, A., Wayne, G., & Danihelka, I. (2014). Neural Turing machines. arXiv:http://arXiv.org/abs/1410.5401

[CR37] Hopfield JJ (1984). Neurons with graded response have collective computational properties like those of two-state neurons. Proceedings of the National Academy of Sciences.

[CR38] Hummel JE (2011). Getting symbols out of a neural architecture. Connection Science.

[CR39] Hupkes D, Veldhoen S, Zuidema W (2018). Visualisation and ’classifiers’ reveal how recurrent and recursive neural networks process hierarchical structure. Journal of Artificial Intelligence Research.

[CR40] Kidd, C., Piantadosi, S.T., & Aslin, R.N. (2012). The goldilocks effect: Human infants allocate attention to visual sequences that are neither too simple nor too complex. *PLOS One* 7 (5). 10.1371/journal.pone.0036399 PMC3359326 22649492

[CR41] Kuehne, S.E., Gentner, D., & Forbus, K.D. (2000). Modeling infant learning via symbolic structural alignment. In *Proceedings of the T,wenty-second Annual Conference of the Cognitive Science Society*, (pp. 286–291).

[CR42] Lake, B., & Baroni, M. (2018). Still not systematic after all these years: On the compositional skills of sequence-to-sequence recurrent networks. *Proceedings of the 35th International Conference on Machine Learning*.

[CR43] Lukoševišius, M. (2012). A practical guide to applying echo state networks. In *Neural Networks: Tricks of the Trade* (pp. 659–686): Springer.

[CR44] Marcus G (1998). Rethinking eliminative connectionism. Cognitive Psychology.

[CR45] Marcus, G. (1999a). Reply to Christiansen and Curtin. *Trends in Cognitive Sciences*, *3*(8), 290–291. ISSN 1364-6613. http://www.sciencedirect.com/science/article/pii/S1364661399013583 10.1016/s1364-6613(99)01358-3 10431182

[CR46] Marcus, G. (1999b). Reply to Seidenberg and Elman. *Trends in Cognitive Sciences*, *3*(8), 288. 10.1016/s1364-6613(99)01357-1 10431181

[CR47] Marcus, G. (1999c). Rule learning by seven-month-old infants and neural networks. Response to Altmann and Dienes. *Science*, *284*, 875. 10.1126/science.283.5398.77 9872745

[CR48] Marcus G, Vijayan S, Rao S, Vishton P (1999). Rule learning by seven-month-old infants. Science.

[CR49] Marcus G (2001). The algebraic mind.

[CR50] Marcus, G. (2018). Deep learning: A critical appraisal. arXiv:1801.00631

[CR51] Mareschal, D., & French, R.M. (1997). A connectionist account of interference effects in early infant memory and categorization. In *Proceedings of the Nineteenth Annual Conference of the Cognitive Science Society*, (pp. 484–489).

[CR52] Marr D (1982). Vision. A computational investigation into the human representation and processing of visual information.

[CR53] McClelland JL, Plaut DC (1999). Does generalization in infant learning implicate abstract algebra-like rules?. Trends in Cognitive Sciences.

[CR54] Miller GA (1956). The magical number seven, plus or minus two: some limits on our capacity for processing information. Psychological Review.

[CR55] Negishi M (1999). Do infants learn grammar with algebra or statistics?. Science.

[CR56] Nelson DGK, Jusczyk PW, Mandel DR, Myers J, Turk A, Gerken L (1995). The head-turn preference procedure for testing auditory perception. Infant Behavior and Development.

[CR57] Opitz B, Hofmann J (2015). Concurrence of rule- and similarity-based mechanisms in artificial grammar learning. Cognitive Psychology.

[CR58] O’Reilly RC (1996). Biologically plausible error-driven learning using local activation differences: The generalized recirculation algorithm. Neural Computation.

[CR59] O’Reilly RC (1998). Six principles for biologically based computational models of cortical cognition. Trends in Cognitive Sciences.

[CR60] Peña M, Bonatti L, Nespor M, Mehler J (2002). Signal-driven computations in speech processing. Science.

[CR61] Pylyshyn Z (1991). Rules and representations: Chomsky and representational realism.

[CR62] Rumelhart DE, Hinton GE, Williams RJ (1988). Learning representations by back-propagating errors. Cognitive Modeling.

[CR63] Saffran, J.R., Aslin, R.N., & Newport, E.L. (1996a). Statistical learning by 8-month-old infants. *Science*, *274*(5294), 1926–1928. 10.1126/science.274.5294.1926 8943209

[CR64] Saffran, J.R., Newport, E.L., & Aslin, R.N. (1996b). Word segmentation: The role of distributional cues. *Journal of Memory and Language*, *35*(4), 606–621.

[CR65] Santoro, A., Bartunov, S., Botvinick, M., Wierstra, D., & Lillicrap, T. (2016). One-shot learning with memory-augmented neural networks. arXiv:1605.06065

[CR66] Seidenberg, M.S., & Elman, J.L. (1999a). Do infants learn grammar with algebra or statistics? *Science*, *284* (5413), 433. 10.1126/science.284.5413.433f 10232987

[CR67] Seidenberg, M.S., & Elman, J.L. (1999b). Networks are not ’hidden rules’. *Trends in Cognitive Sciences*, *3* (8), 288–289. 10.1016/s1364-6613(99)01355-8 10431180

[CR68] Shastri L, Ajjanagadde V, Bonatti L, Lange T, Dyer M (1993). From simple associations to systematic reasoning: A connectionist representation of rules, variables, and dynamic bindings using temporal synchrony. Behavioral and Brain Sciences.

[CR69] Shastri L, Chang S (1999). A spatiotemporal connectionist model of algebraic rule-learning. Technical Report TR-99-011.

[CR70] Shultz, T.R. (1999). Rule learning by habituation can be simulated in neural networks. In *Proceedings of the Twenty-first Annual Conference of the Cognitive Science Society*, (pp. 665–670).

[CR71] Shultz, T.R. (2001). Assessing generalization in connectionist and rule-based models under the learning constraint. In *Proceedings of the Twenty-third Annual Conference of the Cognitive Science Society*.

[CR72] Sirois S, Buckingham D, Shultz TR (2000). Artificial grammar learning by infants: an auto-associator perspective. Developmental Science.

[CR73] Stork, D.G. (1989). Is backpropagation biologically plausible? In *International Joint Conference on Neural Networks*, (Vol. 2 pp. 241–246).

[CR74] Tenenbaum JB, Griffiths TL (2001). Generalization, similarity, and Bayesian inference. Behavioral and Brain Sciences.

[CR75] Tsuji S, Bergmann C, Cristia A (2014). Community-augmented meta-analyses: Toward cumulative data assessment. Perspectives on Psychological Science.

[CR76] Vilcu M, Hadley RF (2005). Two apparent ’counterexamples’ to Marcus: A closer look. Minds and Machines.

[CR77] Wang FH, Zevin JD, Mintz TH (2017). Top-down structure influences learning of nonadjacent dependencies in an artificial language. Journal of Experimental Psychology: General.

[CR78] Woensdregt, M. (2014). Stats & nets versus rules & symbols: Re-opening the debate on learning mechanisms for artificial. Master’s thesis University of Amsterdam.

[CR79] Xie X, Seung HS (2003). Equivalence of backpropagation and contrastive Hebbian learning in a layered network. Neural Computation.

[CR80] Zipser D, Andersen RA (1988). A back-propagation programmed network that simulates response properties of a subset of posterior parietal neurons. Nature.

